# Supplementary material for: The Union Is Strength: The Synergic Action of Long Fatty Acids and a Bacteriophage against Xanthomonas campestris Biofilm
Source: Microorganisms. 2020 Dec 28;9(1):60. doi: 10.3390/microorganisms9010060 (PMC7824728; doi:10.3390/microorganisms9010060)
Supplement: Supplementary file 1 [file microorganisms-09-00060-s001.pdf]

The union is strength: the synergic action of long fatty acids and a bacteriophage against *Xanthomonas campestris* biofilm

Marina Papaianni [1#], Annarita Ricciardelli [2#], Angela Casillo [2], Maria M. Corsaro [2], Fabio Borbone [2], Bartolomeo Della Ventura [3], Raffaele Velotta [3], Andrea Fulgione [4], Sheridan L. Woo [5], Maria L. Tutino [2], Ermenegilda Parrilli [2*], Rosanna Capparelli [1]

[1]Department of Agricultural Sciences, University of Naples Federico II, 80055 Portici, Naples, Italy.
[2]Department of Chemical Sciences, University of Naples Federico II, 80125 Naples, Italy
[3]Department of Physical Sciences, University of Naples Federico II, 80125 Naples, Italy
[4]Istituto Zooprofilattico Sperimentale del Mezzogiorno (IZSM), 80055 Portici, Naples, Italy.
[5]Department of Pharmacy, University of Naples Federico II, 80131 Naples, Italy
[#] These authors contributed equally to this work
*Corresponding Author: Ermenegilda Parrilli email: erparril@unina.it

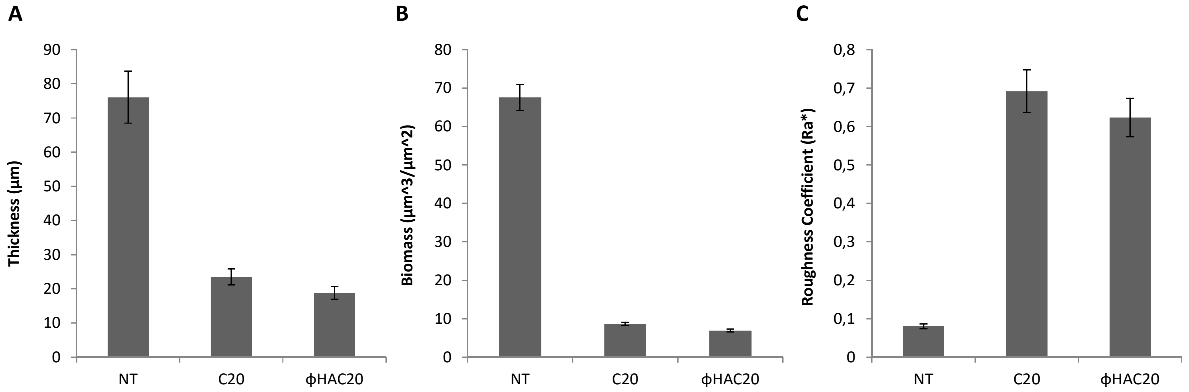

Figure S1 COMSTAT quantitative analysis of average thickness (A), biomass (B) and roughness coefficient (C) of *Xanthomonas campestris* pv. *campestris* biofilms formed in dynamic conditions in absence and in presence of the C20:0 and the complex ϕHA+C20.

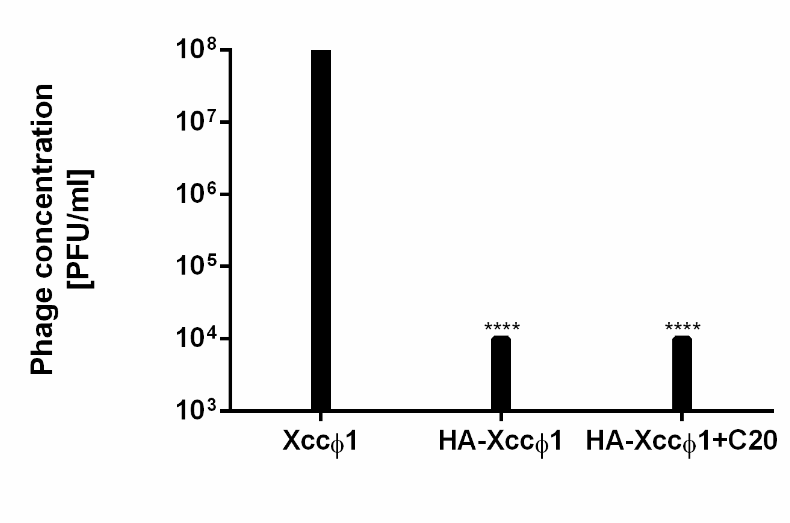

Figure S2 Analysis of the capacity of the HA to binding Xccϕ1 with or without C20:0. Each value is the mean ± DS of 3 independent experiments. Statistical analysis was performed with the absorbance compared to the untreated control and considered statistically significant when $p < 0.05$ (*$p < 0.05$, **$p < 0.01$, ***$p < 0.001$, ****$p<0.0001$) according to Two Way Anova Multiple comparisons.

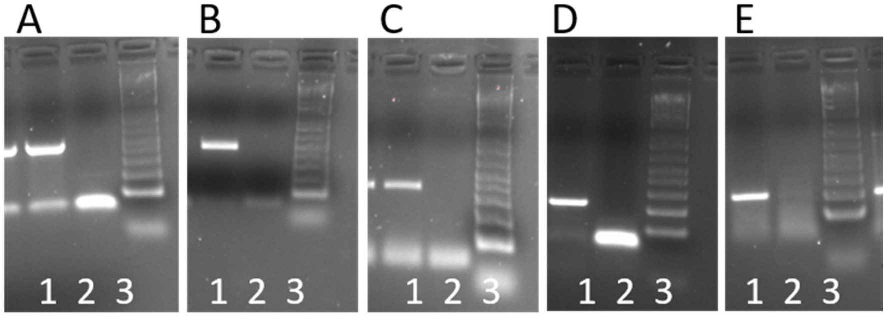

Figure S3 PCR controls. 1) amplification on DNA to assess the primers functionality; 2) amplification using extracted mRNA as template to verify the absence of genomic DNA in the mRNA preparation; 3) Marker (A) *clp*; (B) *man*A; (C) *rpfF*; (D); *rpoD*.

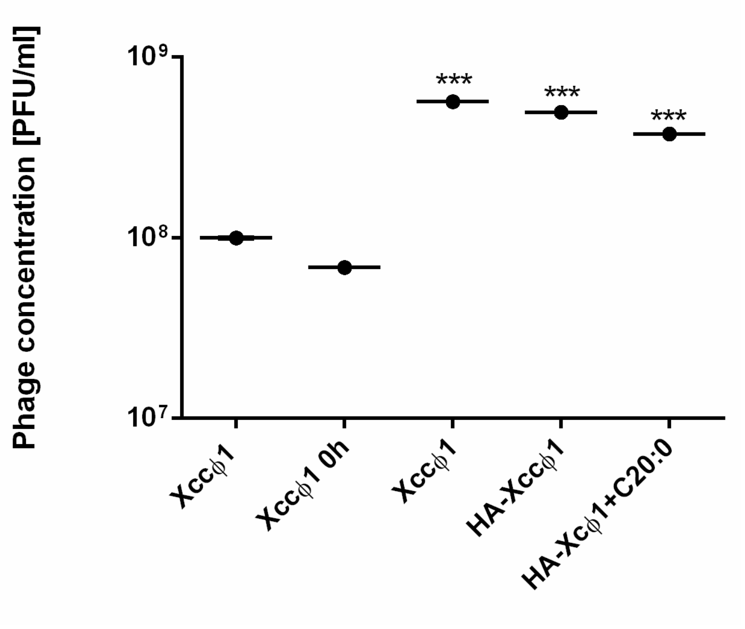

Figure S4 Analysis of phage concentration on the Xcc biofilm before (0h) and after 6h of treatment with: phage alone (108 PFU/mL), the phage with the HA (108 PFU/mL, and 100 mg/mL, respectively) and the complex HA-Xccϕ1 and C20:0. Each value is the mean ± DS of 3 independent experiments. Statistical analysis was performed with the phage concentration after the treatment compared to the phage concentration and considered statistically significant when $p < 0.05$ (*$p < 0.05$, **$p < 0.01$, ***$p < 0.001$, ****$p<0.0001$) according to Two Way Anova Multiple comparisons.
